# The Paradox of Knowledge: Why Medical Students Know More But Understand Less

**DOI:** 10.1007/s40670-025-02379-8

**Published:** 2025-04-14

**Authors:** Heidi L. Lujan, Stephen E. DiCarlo

**Affiliations:** Department of Physiology, College of Osteopathic Medicine, Michigan State University, 567 Wilson Rd. BPS Room 1104, East Lansing, MI 48824 USA

**Keywords:** Thinkers, Problem solvers, Lifelong learners

## Abstract

Medical education faces a paradox: despite unprecedented access to information, many students struggle to apply their knowledge to complex clinical scenarios. This paradox stems from an educational system that prioritizes rote memorization and exam performance over critical thinking and conceptual understanding. Information overload, protocol-driven learning, and exam-focused curricula contribute to superficial learning, leaving students underprepared for real-world practice. Additionally, diminishing emphasis on foundational sciences and inadequate time for reflection exacerbate this issue. To address these concerns, medical education must shift toward fostering deep learning, integrating basic and clinical sciences, and prioritizing active, inquiry-driven teaching methods to prepare adaptable, thoughtful clinicians.

## The Paradox

How is it possible that medical students, armed with unprecedented access to knowledge, often struggle to apply what they have learned in real-world situations? In modern medical education, a striking paradox has emerged: students are bombarded with information, yet many lack the deeper understanding necessary to navigate complex clinical cases. While they can often recite drug protocols and list diagnostic criteria, they frequently miss the “why” behind these facts, leaving them with fragmented knowledge. The current medical education system appears to be producing graduates who are limited in perspective and disheartened, as the curriculum prioritizes the absorption of factual knowledge over the cultivation of critical thinking and problem-solving skills [1]. The problem lies not in their intelligence or dedication, but in an educational system that values and encourages superficial knowledge retention over conceptual comprehension. To understand this paradox, we must explore the forces shaping today’s medical education: from information overload and exam pressures to protocol-driven learning and the reduced emphasis on foundational science.

## The Information Overload in Modern Medicine

Medical students today have access to an almost overwhelming amount of information. With vast online resources, databases, and constantly updated research at their fingertips, it seems as though the sky is the limit for knowledge acquisition. However, this abundance of information has a downside—information overload [2]. While having resources like Up-To-Date, PubMed, and textbooks available on mobile devices allows for rapid access to information, it also leads to superficial learning.

This “Google generation” of medical students may be able to look up any fact in seconds, but quick access does not always translate to meaningful retention. The brain’s capacity to process vast amounts of information at once is limited, and much of what is rapidly consumed is forgotten just as quickly. Cognitive Load Theory (CLT) posits that working memory can only handle a limited amount of information at any given time, creating a bottleneck for learning. When this capacity is exceeded, the efficiency of learning diminishes, leading to rapid forgetting of information. This limitation underscores the importance of instructional designs that manage cognitive load to promote meaningful learning and retention [3–8].

The result of information overload is a population of students who “know” a lot but do not necessarily internalize what they have learned. This superficial learning fails to provide a foundation for the deep understanding necessary to tackle complex clinical problems [9–11].

In this context, medical education has evolved to focus on cramming vast amounts of knowledge into a limited timeframe. The constant push to keep up with expanding fields like genomics, physiology, pharmacology, and evolving evidence-based guidelines often leads students to accumulate broad, surface-level knowledge, while missing the opportunity to deeply master the core principles. Consequently, medical education has turned into a race of data memorization, leaving little room for critical thinking, reflection, and meaningful problem-solving. Medical education must be restructured to prioritize the scientific knowledge essential for navigating today’s complex healthcare system. [12].

## The Pitfalls of Examination-Driven Learning

Examination-driven learning is a deeply embedded aspect of medical education, influencing both early coursework assessments and high-stakes licensing exams [13]. The structure of medical curricula often prioritizes unit and course-level exams early in training, conditioning students to approach learning in a way that prioritizes exam performance over deep understanding. Because these lower-stakes exams shape students’ study habits from the beginning, they reinforce a cycle of rote memorization that persists throughout medical school [14].

As students progress, they encounter major standardized assessments such as the United States Medical Licensing Examination (USMLE) Step 1, and the Comprehensive Osteopathic Medical Licensing Exam (COMLEX-USA) Level 1. These high-stakes exams drive curricula and student study behaviors, as success on these tests plays a critical role in career opportunities, including residency placement. Because course exams often reflect the structure and content of these licensing exams, students begin tailoring their learning strategies early, focusing on test performance rather than conceptual mastery [15].

The sheer volume of information students must master exacerbates this issue. [16, 17]. With a constant cycle of exams, little time is available for reflection or synthesis of knowledge, preventing students from integrating concepts in a meaningful way. Instead, learning becomes fragmented, dictated by exam schedules rather than intellectual curiosity.

Ultimately, the way exams are structured—whether early in coursework or in high-stakes licensure exams—shapes how students study and learn. By restructuring assessments to promote true understanding, medical education can shift from a system of exam-driven knowledge retention to one that nurtures lifelong learners equipped for the complexities of clinical practice [13, 18, 19].

## Traditional Assessments in Medical Education

Traditional assessments in medical education have often emphasized fact-based recall rather than deep understanding. Fact-based multiple-choice questions that focus on isolated drug side effects or biochemical pathways reward rote memorization but may not adequately assess a student’s ability to apply this knowledge in a clinical context. Research has shown that fact-based MCQs, which lack context and problem-solving components, often lead to superficial learning, wherein students can correctly recall isolated details but struggle to integrate and apply this knowledge in real-world medical scenarios [20, 21].

Conversely, clinical vignette-based questions, also known as context-rich or case-based MCQs, present scenarios that require learners to engage in higher-order thinking. These questions assess not just knowledge recall but also application, analysis, and decision-making, which are essential skills for clinical practice. Studies suggest that vignette-based assessments encourage students to develop a deeper understanding of pharmacodynamics and pharmacokinetics, helping them apply these principles when encountering atypical drug reactions or complex patient cases [22, 23].

In addition, medical education has increasingly moved toward integrated curricula and case-based assessments in response to concerns about superficial learning. Integrated curricula aim to connect foundational sciences with clinical practice from the early years of training, helping students see the relevance of their knowledge and apply it meaningfully. This shift has led to improved clinical reasoning skills, as students engage in longitudinal case-based learning rather than discipline-specific memorization [24].

Additionally, the adoption of clinical vignette-based assessments in major standardized exams, such as USMLE, COMLEX-USA, and national board exams worldwide, reflects this pedagogical evolution. The United States Medical Licensing Examination Step 1, for example, has moved away from simple recall questions toward integrated questions that test conceptual application [25]. The shift to Pass/Fail grading for national board exams has further emphasized competency-based assessment over score-based performance, encouraging deeper engagement with material rather than high-stakes memorization [26].

As medical education continues to evolve, assessment strategies must further emphasize clinical reasoning and decision-making. Research suggests that combining progress testing, structured oral examinations, and concept-based MCQs can provide a more accurate measure of a student’s ability to synthesize and apply knowledge [27]. Furthermore, the use of adaptive learning technology in medical assessments can tailor question difficulty based on individual student performance, further promoting deep learning over memorization [28].

Thus, medical educators are redesigning assessments to foster deeper learning and enhance students’ digital literacy skills [29]. Additionally, examination questions are being constructed to assess understanding and integration of content, promoting higher-order learning and producing better practitioners [14]. These changes are essential for improving the quality of medical education and preparing students for the complexities of clinical practice.

## Has Redesigning Assessments and Curricula to Foster Deeper Learning Improved This Issue?

While the integration of curricula, case-based assessments and shift to Pass/Fail exams represents a significant step forward to improving this concern, it has not fully eliminated the challenges of superficial learning. One reason is that simply embedding clinical cases into assessments does not inherently guarantee deeper understanding—it depends on how students interact with the material. Many students, particularly those navigating the pressures of medical school, still default to pattern recognition and memorization rather than engaging in true clinical reasoning. When time is limited, even complex clinical vignettes can become little more than sophisticated multiple-choice exercises, reinforcing test-taking strategies rather than meaningful comprehension.

Additionally, while the Pass/Fail shift for board exams aims to reduce high-stakes memorization, it has introduced new unintended consequences. Without the numerical score as a differentiator, many students now feel increased pressure to excel in other standardized assessments, such as Step 2 CK or specialty board exams—potentially shifting, rather than solving, the problem of rote learning [26, 28, 30]. Furthermore, some medical schools have compensated by increasing internal assessments, ensuring that students still focus on memorization-heavy content to prepare for later stages of training.

The shift toward clinician-led instruction in place of dedicated basic scientists has, in some instances, led to an overreliance on simplified diagnostic and treatment algorithms, prioritizing efficiency over deep conceptual understanding. This phenomenon aligns with concerns in medical education regarding surface learning, where students may perform well on standardized exams yet struggle with applying foundational scientific principles in complex clinical situations. An optimal approach integrates both clinician expertise and foundational science educators to provide a balanced and mechanistic understanding of disease processes. Research suggests that students benefit most from dual-instructor models, where clinicians provide real-world application, while foundational scientists ensure that the underlying principles are not lost in translation [20, 24, 31]. Furthermore, while students often rate algorithm-driven teaching methods highly in evaluations, we acknowledge that high student satisfaction does not always equate to deep learning or clinical competence.

Ultimately, while these reforms have improved aspects of medical education, they have not fully shifted the culture away from memorization. Too many students are being taught to see their value solely in terms of grades, rather than their personal and intellectual growth [32]. Too many students and faculty perceive success only as the score on exams [32]. Until this perspective changes and assessments are designed to truly reward flexible thinking, problem-solving, and lifelong learning skills, students will continue to prioritize performance over deep conceptual mastery.

## Students Are Obsessed with Their Exam Scores

Many students feel driven to perform well on exams not for the sake of learning but because of the weight placed on exam scores by faculty and institutions [32]. The obsession with grades is fueled by a competitive academic environment where test scores significantly influence academic and career outcomes [33]. As faculty prioritize exams in evaluating student performance, students feel compelled to maximize their scores [32]. This environment pushes them toward methods that might ensure short-term recall but discourage long-term understanding. Instead of exploring ideas, making connections, and questioning what they learn, students often reduce learning to a transactional act of retaining information just long enough to perform well on exams [34].

When faculty emphasize exams, they may inadvertently signal to students that scores are the primary measure of success, sidelining critical thinking, creativity, and practical application [32]. This environment undermines students’ motivation to explore knowledge beyond the exam content, narrowing their curiosity and reducing learning to a means to an end. Reframing educational goals to prioritize understanding over rote knowledge could benefit students by encouraging them to engage meaningfully with content, develop genuine intellectual curiosity, and foster a lifelong love of learning. In doing so, we could shift from a model of “knowing more” to one of truly “understanding more,” equipping students not just with answers but with the ability to ask and investigate the right questions [34, 35].

## The Rise of Protocol-Based Medicine

One of the great achievements of modern medicine is the development of protocols and guidelines that standardize care across the globe. These protocols ensure that patients receive evidence-based, reliable treatment, and they have undoubtedly improved patient outcomes in countless scenarios [36]. However, an unintended consequence of this rise in protocol-driven care is that it can lead to algorithmic thinking, where students (and even practicing physicians) follow checklists and guidelines without fully understanding the underlying physiology or pathophysiology that informs these recommendations [37, 38].

For instance, students might be adept at following a sepsis protocol—administering fluids, antibiotics, and vasopressors according to the guidelines—but they may struggle to explain the physiological rationale behind each step. While this method can be highly effective in standard cases, it leaves practitioners vulnerable when faced with atypical scenarios or when the protocol does not perfectly apply to a patient’s unique condition. Understanding the “why” behind the protocol is essential for adapting care to complex, non-standard cases, but when students rely too heavily on pre-established guidelines, this deeper understanding may be sacrificed.

This reliance on protocols has also led to a diminished focus on foundational sciences. Physiology, biochemistry, and pathophysiology, once cornerstones of medical education, are sometimes perceived as less important compared to clinical guidelines and treatment algorithms. As a result, students may not spend as much time exploring the principles that underlie medical care, leading to a situation where they know what to do, but not why they are doing it [37, 38].

## Are Students “Successful But Unwise”?

Given the challenges faced by modern medical students, it might be tempting to label them as “successful but unwise”—a phrase suggesting that they excel at absorbing vast amounts of information without understanding it deeply. However, this label oversimplifies the issue and unfairly criticizes students for a systemic problem that exists in medical education today.

A more accurate description might be to view medical students as “overburdened learners.” They are intelligent, hardworking individuals navigating an educational system that prioritizes knowledge acquisition over conceptual understanding. The problem is not that they lack the ability to think critically or understand deeply, but that the system is structured in such a way that they rarely have the time or opportunity to do so [12, 29].

Medical students today are being asked to juggle an overwhelming amount of material, often at the expense of developing a deeper, more integrated understanding of the content. They excel in exams and pass rigorous academic hurdles, but the constant demand to move quickly from one topic to the next can leave them feeling as if they have only skimmed the surface of what they need to know. Additionally, the increasing use of digital resources requires new literacy skills for effective learning [29]. This is not a reflection of their intelligence or capability, but of the systemic pressures they face.

## The Shift Toward Clinical Learning and Away from Fundamental Science

In the past decades, medical education has increasingly emphasized early clinical exposure and practical skills [39, 40]. This shift is meant to help students apply their learning to real-world scenarios more quickly and prepare them for the hands-on nature of medical practice. While early clinical exposure is undoubtedly valuable, it can sometimes come at the expense of focusing on the basic sciences that are critical for long-term understanding [41].

Medical students are often placed into clinical rotations before they have had a chance to fully digest the mechanisms of disease or the fundamentals of physiology. In the rush to develop clinical competencies, there is less time to dwell on the nuances of biochemical pathways, immunological responses, or the detailed anatomy of structures being treated [24, 39, 41–43]. These foundational sciences are the very principles that allow physicians to think critically about complex cases.

This approach can lead to students developing practical skills without context [40]. They may become proficient at drawing blood, suturing wounds, or ordering tests, but they may not fully grasp why they are doing these things, what the results mean, or how it all fits into the broader context of patient care [40].

The foundational role of basic sciences in developing critical thinking, problem-solving, and scientific curiosity must be noted [37, 39]. However, the increasing clinical knowledge and time constraints in curricula pose challenges to adequate basic science education [37, 42]. Despite these challenges, understanding basic sciences remains essential for clinical practice and should be integrated with clinical applications throughout medical education [24, 42].

## The Lack of Time for Reflection and Synthesis

Understanding in medicine often comes not from merely acquiring knowledge, but from the ability to synthesize information and apply it in various contexts [17, 44]. This requires time for reflection, integration, and problem-solving [25, 45]. However, the modern medical curriculum is often so packed with information that students rarely have the opportunity to think critically about what they are learning [16].

Reflective learning is crucial for making connections between disparate pieces of knowledge. For example, understanding how cardiovascular physiology relates to the treatment of hypertension or how immune system dysregulation can influence the development of autoimmune diseases requires time to step back and draw these connections [46–49]. Without opportunities for reflection, students may memorize facts independently but fail to integrate them into a cohesive understanding, thereby missing out on richer learning experiences.

## The Role of Teaching in Developing Understanding

The way material is taught can greatly influence whether students develop a deep understanding. Didactic teaching methods, where an instructor simply lectures students on facts, can lead to passive learning. In contrast, active learning methods, such as case-based discussions, interactive problem-solving, and clinical reasoning exercises, can help students engage more deeply with the material [50].

Instructors who encourage critical thinking and ask students to explain the reasoning behind diagnoses or treatment decisions promote a learning environment where understanding is valued over memorization [51]. Thus, teaching should be a process that encourages inquiry, rather than simply delivering information for students to absorb. This can only occur with face-to-face instruction [51]. Accordingly, the increased reliance on prerecorded lectures as a source of learning in place of live lectures in higher education must be reversed.

## Moving from Knowing to Understanding: The Way Forward

In today’s academic landscape, the relentless pursuit of exam performance often eclipses the deeper quest for understanding. Students, driven by the pressures of grades and future prospects, focus on mastering test-taking strategies rather than cultivating critical thinking and genuine curiosity. Faculty, too, find themselves trapped in this cycle, designing curricula tailored to standardized assessments rather than fostering environments that nurture intellectual exploration.

Meaningful change demands a collective shift—one that involves students valuing knowledge over mere credentials, educators embodying the curiosity and wisdom they hope to inspire, and institutions reevaluating success beyond test scores [32, 52]. A truly transformative education system would reward curiosity, encourage intellectual risk-taking, and foster the development of lifelong learners. Until this triad of change occurs, the cycle will persist, leaving generations of students well-prepared for exams but ill-equipped for the complex realities beyond the classroom.

## References

[1] Witt EE, Onorato SE, Schwartzstein RM. Medical students and the drive for a single right answer: teaching complexity and uncertainty. ATS Sch. 2022;3(1):27–37. 35633993 10.34197/ats-scholar.2021-0083PS PMC9131886

[2] Achike FI, Ogle CW. Information overload in the teaching of pharmacology. J Clin Pharmacol. 2000;40(2):177–83. 10664924 10.1177/00912700022008838

[3] Sweller J. Cognitive load during problem solving: effects on learning. Cogn Sci. 1988;12(2):257–85.

[4] Sweller J. Cognitive load theory. In: Seel NM, editor. Encyclopedia of the sciences of learning. Boston, MA: Springer, US; 2012. p. 601–5.

[5] van Merriënboer JJG, Sweller J. Cognitive load theory and complex learning: recent developments and future directions. Educ Psychol Rev. 2005;17(2):147–77.

[6] Paas F, Renkl A, Sweller J. Cognitive load theory and instructional design: recent developments. Educ Psychol. 2003;38(1):1–4.

[7] Miller GA. The magical number seven plus or minus two: some limits on our capacity for processing information. Psychol Rev. 1956;63(2):81–97. 13310704

[8] Young JQ, Van Merrienboer J, Durning S, Ten Cate O. Cognitive load theory: implications for medical education: AMEE Guide No. 86. Med Teach. 2014;36(5):371–84. 24593808 10.3109/0142159X.2014.889290

[9] Dolmans D, Loyens SMM, Marcq H, Gijbels D. Deep and surface learning in problem-based learning: a review of the literature. Adv Health Sci Educ Theory Pract. 2016;21(5):1087–112. 26563722 10.1007/s10459-015-9645-6 PMC5119847

[10] Entwistle NJ, Peterson ER. Conceptions of learning and knowledge in higher education: relationships with study behaviour and influences of learning environments. Int J Educ Res. 2004;41(6):407–28.

[11] Neumann DL, Hood M. The effects of using a wiki on student engagement and learning of report writing skills in a university statistics course. Australas J Educ Technol. 2009;25(3).

[12] Lucey CR. Medical education: part of the problem and part of the solution. JAMA Intern Med. 2013;173(17):1639–43. 23857567 10.1001/jamainternmed.2013.9074

[13] Lujan HL, DiCarlo SE. Beyond the boards: too much time in study hall, too little societal impact. Adv Physiol Educ. 2024;48(4):756–8. 39116393 10.1152/advan.00148.2024

[14] Burns ER. “Anatomizing” reversed: use of examination questions that foster use of higher order learning skills by students. Anat Sci Educ. 2010;3(6):330–4. 21046570 10.1002/ase.187

[15] Artino AR Jr, Dong T, DeZee KJ, Gilliland WR, Waechter DM, Cruess D, et al. Achievement goal structures and self-regulated learning: relationships and changes in medical school. Acad Med: J Assoc Am Med Coll. 2012;87(10):1375–81. 10.1097/ACM.0b013e3182676b55 22914521

[16] DiCarlo SE. Cell biology should be taught as science is practised. Nat Rev Mol Cell Biol. 2006;7(4):290–6. 16482092 10.1038/nrm1856

[17] DiCarlo SE. Too much content, not enough thinking, and too little FUN! Adv Physiol Educ. 2009;33(4):257–64. 19948670 10.1152/advan.00075.2009

[18] Kassam A, de Vries I, Zabar S, Durning SJ, Holmboe E, Hodges B, et al. The next era of assessment within medical education: exploring intersections of context and implementation. Perspect Med Educ. 2024;13(1):496–506. 39399409 10.5334/pme.1128 PMC11469546

[19] Hafferty FW, O’Brien BC, Tilburt JC. Beyond high-stakes testing: learner trust, educational commodification, and the loss of medical school professionalism. Acad Med: J Assoc Am Med Coll. 2020;95(6):833–7. 10.1097/ACM.0000000000003193 32079955

[20] Norman G. Teaching basic science to optimize transfer. Med Teach. 2009;31(9):807–11. 19811185 10.1080/01421590903049814

[21] Schuwirth LW, Van der Vleuten CP. Programmatic assessment: from assessment of learning to assessment for learning. Med Teach. 2011;33(6):478–85. 21609177 10.3109/0142159X.2011.565828

[22] Schmidt HG, Mamede S. How to improve the teaching of clinical reasoning: a narrative review and a proposal. Med Educ. 2015;49(10):961–73. 26383068 10.1111/medu.12775

[23] Custers E. Long-term retention of basic science knowledge: a review study. Adv Health Sci Educ Theory Pract. 2010;15(1):109–28. 18274876 10.1007/s10459-008-9101-y

[24] Kulasegaram KM, Martimianakis MA, Mylopoulos M, Whitehead CR, Woods NN. Cognition before curriculum: rethinking the integration of basic science and clinical learning. Acad Med: J Assoc Am Med Coll. 2013;88(10):1578–85. 10.1097/ACM.0b013e3182a45def 23969375

[25] Prober CG, Heath C. Lecture halls without lectures–a proposal for medical education. N Engl J Med. 2012;366(18):1657–9. 22551125 10.1056/NEJMp1202451

[26] Rothka AJ, Nguyen M, King TS, Choi KY. Impact of USMLE pass/fail step 1 scoring on current medical students. J Med Educ Curric Dev. 2024;11:23821205241281650. 39346123 10.1177/23821205241281650 PMC11437557

[27] Uijtdehaage S, Schuwirth LWT. Assuring the quality of programmatic assessment: moving beyond psychometrics. Perspect Med Educ. 2018;7(6):350–1. 30406905 10.1007/s40037-018-0485-y PMC6283782

[28] Sharma N, Doherty I, Dong C. Adaptive learning in medical education: the final piece of technology enhanced learning? Ulst Med J. 2017;86(3):198–200. PMC5849979 29581634

[29] Goh PS, Sandars J. Response to: response to: increasing tensions in the ubiquitous use of technology for medical education. Med Teach. 2019;41(10):1212. 30994015 10.1080/0142159X.2019.1595369

[30] Spring L, Robillard D, Gehlbach L, Simas TA. Impact of pass/fail grading on medical students’ well-being and academic outcomes. Med Educ. 2011;45(9):867–77. 21848714 10.1111/j.1365-2923.2011.03989.x

[31] Jiang D, Huang D, Wan H, Fu W, Shi W, Li J, et al. Effect of integrated case-based and problem-based learning on clinical thinking skills of assistant general practitioner trainees: a randomized controlled trial. BMC Med Educ. 2025;25(1):62. 39806361 10.1186/s12909-025-06634-9 PMC11731422

[32] Lujan HL, DiCarlo SE. Students are more than their scores: educators have the power to change how students perceive success. Adv Physiol Educ. 2025;49(1):93-95. 10.1152/advan.00185.2024 39602231

[33] Peterson TA, Clemente R. Failing while making all “A’s”. J High Educ Theory Pract. 2022;22(15):1-11.

[34] Ghaleb BDS. Effect of exam-focused and teacher-centered education systems on students' cognitive and psychological competencies. Int J Multidiscip Approach Res Sci. 2024;2(2):611-631.

[35] Mattick K, Knight L. High-quality learning: harder to achieve than we think? Med Educ. 2007;41(7):638–44. 17614883 10.1111/j.1365-2923.2007.02783.x

[36] Morris AH. Treatment algorithms and protocolized care. Curr Opin Crit Care. 2003;9(3):236–40. 12771677 10.1097/00075198-200306000-00012

[37] Ravesloot JH. [Current medical curricula neglect the basic sciences]. Ned Tijdschr Geneeskund. 2018;Aug 30(162):D3013. 30212019

[38] Terry PD, Knight M, Bollig R, Heidel RE, Miller P, Quinn MA, et al. Overreliance on standardized protocols: a pilot study of surgical residents and fellows. Am Surg. 2017;83(5):e159–61. 28541842

[39] Finnerty EP, Chauvin S, Bonaminio G, Andrews M, Carroll RG, Pangaro LN. Flexner revisited: the role and value of the basic sciences in medical education. Acad Med: J Assoc Am Med Coll. 2010;85(2):349–55. 10.1097/ACM.0b013e3181c88b09 20107367

[40] Finnerty EP. COMMENTARY: why the basic sciences? JIAMSE. 2002;12(1):4.

[41] Woods NN, Brooks LR, Norman GR. The value of basic science in clinical diagnosis: creating coherence among signs and symptoms. Med Educ. 2005;39(1):107–12. 15612907 10.1111/j.1365-2929.2004.02036.x

[42] Bandiera G, Boucher A, Neville A, Kuper A, Hodges B. Integration and timing of basic and clinical sciences education. Med Teach. 2013;35(5):381–7. 23444888 10.3109/0142159X.2013.769674

[43] Harris P, Snell L, Talbot M, Harden RM. Competency-based medical education: implications for undergraduate programs. Med Teach. 2010;32(8):646–50. 20662575 10.3109/0142159X.2010.500703

[44] Lujan HL, DiCarlo SE. Too much teaching, not enough learning: what is the solution? Adv Physiol Educ. 2006;30(1):17–22. 16481604 10.1152/advan.00061.2005

[45] D’Eon M, Kosmas C, MacMillan J. Teaching syndromes–a response to learning syndromes. Med Teach. 2007;29(2–3):280–1 (**discussion 1–2**). 17701648 10.1080/01421590701252115

[46] Sandars J. The use of reflection in medical education: AMEE Guide No. 44. Med Teach. 2009;31(8):685–95. 19811204 10.1080/01421590903050374

[47] Nguyen QD, Fernandez N, Karsenti T, Charlin B. What is reflection? A conceptual analysis of major definitions and a proposal of a five-component model. Med Educ. 2014;48(12):1176–89. 25413911 10.1111/medu.12583

[48] Wald HS, Borkan JM, Taylor JS, Anthony D, Reis SP. Fostering and evaluating reflective capacity in medical education: developing the REFLECT rubric for assessing reflective writing. Acad Med: J Assoc Am Med Coll. 2012;87(1):41–50. 10.1097/ACM.0b013e31823b55fa 22104060

[49] Mamede S, Schmidt HG. The structure of reflective practice in medicine. Med Educ. 2004;38(12):1302–8. 15566542 10.1111/j.1365-2929.2004.01917.x

[50] Wagner FL, Sudacka M, Kononowicz AA, Elvén M, Durning SJ, Hege I, et al. Current status and ongoing needs for the teaching and assessment of clinical reasoning - an international mixed-methods study from the students` and teachers` perspective. BMC Med Educ. 2024;24(1):622. 38840110 10.1186/s12909-024-05518-8 PMC11151606

[51] Lujan HL, Raizada A, DiCarlo SE. Critical skill of teaching: learning the cognitive and emotional states of our students during class. Adv Physiol Educ. 2021;45(1):59–60. 33464192 10.1152/advan.00219.2020

[52] Lujan HL, DiCarlo SE. We used to get money to teach students, now we teach students to get money: medical education has become a market with credentials not knowledge the commodity! Adv Physiol Educ. 2023;47(3):521–6. 37262109 10.1152/advan.00065.2023

